# Delayed feeding disrupts diurnal oscillations in the gut microbiome of a neotropical bat in captivity

**DOI:** 10.1093/femsec/fiaf012

**Published:** 2025-01-22

**Authors:** Dominik W Melville, Magdalena Meyer, Corbinian Kümmerle, Kevin A Alvarado-Barrantes, Kerstin Wilhelm, Simone Sommer, Marco Tschapka, Alice Risely

**Affiliations:** Institute of Evolutionary Ecology and Conservation Genomics, Ulm University, 89081 Ulm, Germany; Institute of Evolutionary Ecology and Conservation Genomics, Ulm University, 89081 Ulm, Germany; Institute of Evolutionary Ecology and Conservation Genomics, Ulm University, 89081 Ulm, Germany; Escuela de Biología, Universidad de Costa Rica, 2060 San Jose, Costa Rica; Institute of Evolutionary Ecology and Conservation Genomics, Ulm University, 89081 Ulm, Germany; Institute of Evolutionary Ecology and Conservation Genomics, Ulm University, 89081 Ulm, Germany; Institute of Evolutionary Ecology and Conservation Genomics, Ulm University, 89081 Ulm, Germany; School of Science, Engineering and Environment, Salford University, M5 4WT Manchester, United Kingdom

**Keywords:** circadian rhythm, diet experiment, dysbiosis, gut pH, *Leptonycteris yerbabuenae*, microbial ecology

## Abstract

Diurnal rhythms of the gut microbiota are emerging as an important yet often overlooked facet of microbial ecology. Feeding is thought to stimulate gut microbial rhythmicity, but this has not been explicitly tested. Moreover, the role of the gut environment is entirely unexplored, with rhythmic changes to gut pH rather than feeding *per se* possibly affecting gut microbial fluctuations. In this study, we experimentally manipulated the feeding schedule of captive lesser long-nosed bats, *Leptonycteris yerbabuenae*, to dissociate photic and feeding cues, and measured the faecal microbiota and gut pH every 2 h. We detected strong diurnal rhythms in both microbial alpha diversity and beta diversity as well as in pH within the control group. However, a delay in feeding disrupted oscillations of gut microbial diversity and composition, but did not affect rhythms in gut pH. The oscillations of some genera, such as *Streptococcus*, which aid in metabolizing nutrients, shifted in accordance with the delayed-feeding cue and were correlated with pH. For other bacterial genera, oscillations were disturbed and no connection to pH was found. Our findings suggest that the rhythmic proliferation of bacteria matches peak feeding times, providing evidence that diurnal rhythms of the gut microbiota likely evolved to optimize their metabolic support to the host’s circadian phenotype.

## Teaser

The study shows that feeding stimulates rhythmicity in the gut microbial community over a 24-h period, which has consequences for how the microbiome mediates host metabolic and immunological functions.

## Introduction

Circadian rhythms coordinate bio-chemo-physical processes over a 24-h period (Yerushalmi and Green 2009). While these rhythms are self-sustained, they are entrained by environmental cues, i.e. zeitgeber, to meet diurnally recurring challenges. Photic cues entrain the master pacemaker located in the brain, whereas nonphotic cues largely synchronize peripheral clocks in organs and tissues (Bell-Pedersen et al. 2005, Buhr et al. 2010, Lewis et al. 2020, Segers and Depoortere 2021). Mounting evidence points towards feeding being fundamental for orchestrating system-wide physiological homeostasis in immunity and metabolism throughout the day (Thaiss et al. 2014, 2016, Kaczmarek et al. 2017, Teichman et al. 2020, Tognini et al. 2020, Tuganbaev et al. 2020, Brooks et al. 2021). The gut microbiome—a diverse set of microbes and their metabolic products—is thought of as an important intermediary between feeding cues and physiological response (Sommer et al. 2016, Frazier and Chang 2020). Particularly telling is that the absence of gut microbiota dampens circadian expression of central and peripheral clock genes, even when light and feeding cues are present (Leone et al. 2015). Hence, the circadian phenotype is a product of host and microbiome-mediated processes. And yet, the oscillation of gut microbiota throughout the day and the cues that maintain them have been notably overlooked in the ecology and evolution of host-associated microbiomes (Schmid et al. 2023).

Circadian rhythms have been identified for a variety of different host-associated microbial communities [e.g. corals (Rosenberg et al. 2022); flatworms (Ma et al. 2023)]. However, diurnal rhythms in the gut microbial community, which are most intimately linked to host physiology, immunity, and behaviour, were only reported in a few natural (e.g. meerkats, hyenas, warblers, and humans; Reitmeier et al. 2020, Risely et al. 2021, Melville et al. 2024, Worsley et al. 2024a) and captive populations (e.g. mice, chickens, and fish; Hieke et al. 2019, Parris et al. 2019, Brooks et al. 2021, Allaband et al. 2024). Between ∼10% and 40% of resident gut microbes are estimated to oscillate (Thaiss et al. 2014, Zarrinpar et al. 2014, Reitmeier et al. 2020), and this translates into functionally important rhythmicity in transcriptomes, metabolites, and gene content over 24 h (Leone et al. 2015, Thaiss et al. 2016, Kaczmarek et al. 2017). Feeding cues are handled as an important cue for the gut microbiota, and many oscillating taxa are thought to play key roles in assimilating nutrients from food (Brooks et al. 2021, Risely et al. 2021). In addition, competition by and metabolic products of bacteria may also periodically alter the abiotic and biotic gut environment, but the ecological niche ‘gut’ is rarely considered. For instance, the gut pH of ruminants undergoes concurrent changes as the gut microbial community shifts after feeding (Shaani et al. 2018). Yet, aside from hints, we lack experimental evidence of the cues that initiate and maintain circadian rhythms in the gut.

Bats are the second most speciose mammalian order and occupy diverse ecological niches. Their phylogenetic and ecological diversity, as well as some of their biological peculiarities (e.g. longevity), make them ideal nonmodel organisms to tackle some fundamental questions in microbiome ecology and evolution (Ingala et al. 2018). For one, gut microbial communities are adapted to the diverse feeding niches bats occupy (Carrillo-Araujo et al. 2015, Phillips et al. 2017, Zepeda Mendoza et al. 2018, Lutz et al. 2019, Ingala et al. 2021), and respond readily to diet changes across seasons (Gong et al. 2021, Víquez-R et al. 2021) and between landscapes (Ingala et al. 2019, Fleischer et al. 2024), while maintaining core bacterial taxa. This implies resident gut bacteria are tasked with certain metabolic functions (Phillips et al. 2017, Zepeda Mendoza et al. 2018). Furthermore, short gut transit times in bats circumvent a problem found in other species, where sectional gut morphology and lengthy gut transit times confound the relationship between food intake and gut microbial dynamics (Carrillo-Araujo et al. 2015). The presence of diurnal gut microbial rhythms synchronized to the host’s ecological demands would underscore the importance of microbiota in bat immunity and metabolism.

**Figure 1.**
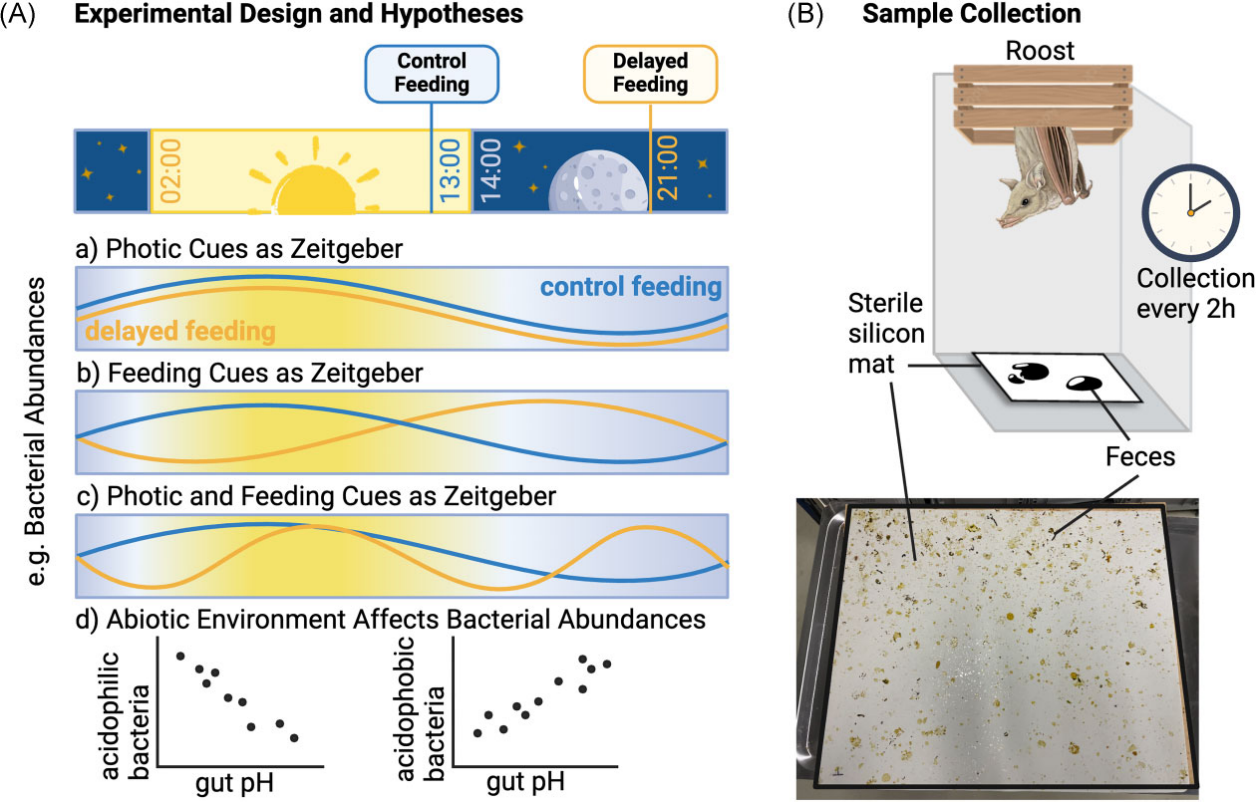
Study design and sampling. (A) Schematic representation of the experimental design, in which the control group experienced their regular light–dark cycle and unchanged feeding times (in blue), while the treatment group’s feeding time was delayed by 8 h (in yellow). If diurnal microbial rhythms were present in the control group, then a delay in feeding might a) have no effect on gut microbial rhythms; b) cause a phase shift; or c) disrupt microbial rhythmicity. We also predict that whether microbiota composition was linked to abiotic conditions in the gut, then bacterial abundances might correlate with pH according to their pH tolerance. (B) Sample collection was completed noninvasively using sterile silicon mats placed underneath bat roosting spots. The mats were recovered every 2 h, and exchanged with clean ones, while bat droppings were collected. Created with BioRender.com.

In this study, we aimed to determine whether gut microbial rhythms can be detected in captive nectivorous lesser long-nosed bats (*Leptonycteris yerbabuenae*, Phyllostomidae: Glossophaginae), and to experimentally test whether delaying feeding times predictably alter gut microbial rhythms. We conducted the experiment on a population of 41 bats attuned to a 12:12 light–dark cycle and fed once a day timed to coincide with the onset of the dark cycle, which is the natural active period in these nectarivores bats (Rivera-Villanueva et al. 2024). We divided the population into two separate groups and delayed the feeding time in the treatment group by 8 h (Fig. 1A). We then noninvasively sampled bat droppings every 2 h over a 48-h period to quantify microbial composition and faecal pH (Fig. 1B). Specifically, we test (i) the effect of an 8-h delay on the diurnal oscillations of the faecal microbiome and pH, and identify (ii) whether any observed changes to rhythms in gut pH mirror those of the microbiome. Assuming that the control group demonstrates diurnal oscillations in the faecal microbial community, we hypothesized (Fig. 1A) (i) that a delay in feeding will have no effect on microbial rhythms, if feeding played no role in synchronizing gut microbial rhythms; however, (ii) if feeding was the main cue shaping microbial rhythms, then microbial rhythms should demonstrate a phase shift; and (iii) if microbial rhythms were synchronized interactively by feeding and other circadian cues (e.g. photic or host genetics), then microbial rhythms should be dampened or disrupted. Lastly, we hypothesized that if gut microbiota oscillations were directly linked to changes in gut abiotic conditions, then changes in microbial rhythms should be matched by similar changes in rhythms in gut pH and a correlational link between the abundance of certain bacteria and pH might be apparent.

## Materials and methods

### Experimental design

The experimental design was preregistered (Schmid et al. 2022). The study population of *L. yerbabuenae* was originally established in 1987 at the University of Erlangen—Nürnberg, Germany, from 15 wild individuals imported from Central Mexico. Some descendants of this initial group are now housed at the University of Ulm, Germany. For the study, the population was divided into two rooms with roughly equal numbers (*n* = 20/21). The bats were adapted to a 12:12 dark–light cycle switching from light to dark at 2 p.m., and are being fed daily at 1 p.m. solutions of pollen, Nektar-Plus™ (Nekton GmbH, Keltern, Germany) and milk powder (Alete™) in honey water (17%–18%; Fig. 1A). The feeding is scheduled this way so that food is freshly available when the dark cycle and, thus, the bats’ active period begins. Each enclosure contained three hanging crates where bats roost.

The experiment was based on a two-group treatment design, consisting of one control and one manipulated group (Fig. 1A), and was run over the course of 2 days (i.e. a total of 48 h) in August 2022. The control group experienced no changes in feeding time, whilst we delayed feeding in the treatment group by 8 h (i.e. at 9 p.m.). Bats in the delayed-feeding treatment group thus received food 8 h later than the control group, and 7 h after the start of their active period. The light–dark cycle was kept constant for both groups, ensuring the only change experienced by bats was an 8-h delay in feeding in the treatment group. Owing to the high metabolic demands of nectar-feeding bats, this was the maximum delay we were willing to tolerate and still allow for a 5-h feeding window before the lights were switched on again.

### Noninvasive faecal sample collection

To generate high-resolution temporal data on faecal microbiome cycles, faecal samples were collected at the end of each 2-h sampling window over the study period. We collected droppings from each enclosure using an entirely noninvasive sampling protocol (Fig. 1B). We placed three sterile silicone mats underneath each roosting spot in each enclosure, starting at 9 a.m. on the first day. Every 2 h, the mats were retrieved and new sterile mats were laid out. After mat retrieval, all distinct droppings were carefully collected using sterile cotton swabs, stored in an Eppendorf vial and immediately frozen at −80°C. The procedure of mat retrieval and faecal sample collection was repeated every 2 h for 48 h. Feed provision was directly after the mats were collected at 1 p.m. and 9 p.m. for the control and delayed-feeding group, respectively, meaning that these collection time points still represented microbial diversity during fasting. After the collection of all droppings, mats were rinsed thoroughly, sterilized with antibacterial soap, and dried.

The noninvasive sample collection meant that we were unable to determine precisely which individual defecated. However, the number of faecal samples collected at every 2-h interval were fewer than the total number of bats in each enclosure (Fig. 1B). Therefore, it is unlikely that samples collected within a 2-h period belong to the same host.

### Measuring faecal pH

We measured pH of each sample as a proxy for gut biochemical conditions. To estimate faecal pH, we first weighed faecal samples. Based on the weight, we added water to reach a standardized 1:10 dilution (Shen et al. 2011). We then homogenized the solution using the sterile tip of a spatula to break up the faecal mass and subsequently vortexed the sample for 10 s, before storing each sample in the fridge. After the coarse material had settled, we used a pH meter (METTLER TOLEDO, USA) to determine the pH of each sample, while rinsing the pH meter first with water and then with ethanol between each sample. The volume of 45 samples (out of 179 samples) was too small to accurately determine their pH.

### 16S rRNA gene metabarcoding

For sequencing the hypervariable V4 region of the 16S rRNA gene, we followed a protocol previously applied to faecal samples from different bat species (Wasimuddin et al. 2018, Alpízar et al. 2021, Fleischer et al. 2022, Melville et al. 2024) and *L. yerbabuenae* (Víquez-R et al. 2021). First, we used the residue from the previous homogenization step after carefully pipetting the supernatant, and proceeded to extract the bacterial DNA using the NucleoSpin 96 Soil kit (Macherey-Nagel, Germany) from 213 samples, aiming at five samples per time point per sampling day (Fig. S1) and including six extraction controls. We amplified the 291 bp V4 region using the primer pair 515F (5′-GTGCCAGCMGCCGCGGTAA-3′) and 806R (5′-GGACTACHVGGGTWTCTAAT-3′). We followed the Fluidigm protocol (Access Array systems for Illumina sequencing, Fluidigm Corporation) for primer tagging. The polymerase chain reaction analysis (15 µl of volume) was performed as described in detail by Menke et al. (2014). Barcoded samples were then purified (NucleoMag bead-based size selection; Macherey-Nagel) and quantified (DropSense, Trinean, USA), before the pooled sample library was paired-end sequenced in a single run on an Illumina MiSeq platform.

### Bioinformatics

Initial sequencing read processing was done using QIIME 2 (v2021.8.0; Bolyen et al. 2019). Following the standard protocol, we removed low quality sequences, trimmed primers, and truncated our forward and reverse reads to 215 and 235 bp, respectively. We applied the DADA2 algorithm for clustering into amplicon sequence variants (ASVs) and denoising (Callahan et al. 2016). We built a phylogenetic tree employing MAFFT (Katoh and Standley 2013) and FastTree (Price et al. 2010), and rooted it using an archaeon sequence (accession number: KU656649). ASVs were taxonomically assigned using the SILVA database (v138; Quast et al. 2012). We filtered out sequences described as archaea or eukaryotes. After this initial filtering, 8 764 203 reads and 1989 unique ASVs remained. The sample meta information, taxonomy table, read counts, and rooted tree were then imported into R (v4.2.1; R Core Team 2022) using the *phyloseq* package (v1.42.0; McMurdie and Holmes 2013). We then filtered out ASVs unclassified at the phylum level or classified as chloroplast, which represent pollen found in their feces rather than gut bacteria. After this step 5 737 770 reads and 1348 unique ASVs remained. Next we filtered out ASVs with fewer than 10 reads in total, phyla with a prevalence below 0.01%, and excluded three low-abundance ASVs identified by the decontamination workflow of the package ‘decontam’ (Davis et al. 2018) to be more frequent in the extraction blanks than in faecal samples. The filtering of rare taxa had minimal impact on sample-level diversity [i.e. rare ASVs removed: mean number of ASVs per sample = 15.8 (±11.5 SD); rare ASVs not removed: mean number of ASVs per sample = 17.8 (±14.1 SD)]. Lastly, 24 samples that had fewer than 500 reads were removed (Fig. S1). The final microbiome data from 179 samples included 5 636 274 reads (maximum: 113 390 reads; minimum: 546 reads; and average: 31 488 reads ± 23 490 reads standard deviation) and 698 AVSs.

### Statistical analysis

Because diurnal oscillations are nonlinear in nature, our approach across all analyses is to apply generalized additive models (GAMs) to identify the effect of feeding delay on the diurnal oscillations in alpha diversity, beta diversity, and the relative abundances of common genera. Since alpha diversity, beta diversity, and relative abundances of specific genera make up distinct data types with different distributions and different sensitivities to read depth and compositionality, the normalization technique for each analysis was chosen based on these factors (Boshuizen and Te Beest 2023). Alpha-diversity data were not normalized but modelled using raw diversity data, data for beta-diversity analyses were normalized through rarefaction, and genus-level abundances were normalized via centred-log ratio (CLR) transformation, because they are compositional. Read depth and sampling day (1 or 2) were controlled for in all models. This approach is consistent with best practice (Baniel et al. 2021, Grieneisen et al. 2021, Bates et al. 2022). We provide more details for each analysis below.

*Microbiome alpha diversity*: We first calculated two alpha-diversity indices, i.e. observed ASVs and Shannon diversity, from unrarefied reads using the *phyloseq::estimate_richnes s*() function. Observed ASVs strictly count the number of distinct ASVs, whereas Shannon diversity considers richness but weighs it according to evenness. Mean observed ASV diversity was low (mean 17.7 ±11.2 SD), and therefore rarefaction curves plateaued at low sequencing depths for the vast majority of samples (Fig. S2). Data rarefied to the minimum read count (i.e. 546) and unrarefied alpha-diversity metrics were highly correlated (*R*^2^ = 81.6, *P* < .01; Fig. S3), but rarefied measures of alpha diversity were still overall lower than unrarefied alpha diversity, therefore significantly underestimating AVS diversity, even whilst still accurately reflecting relative differences in diversity across individuals and treatments. Because the analysis with rarefied and unrarefied data yielded almost identical results (Fig. S4 and Table S1), we opted to report the results of unrarefied alpha diversity (McMurdie and Holmes 2014, Weiss et al. 2017), and accounted for sequencing depth in models.

To model alpha diversity across time, we fitted two GAMs using the *gam*() function of the ‘mgcv’ package (Wood 2017) on each alpha-diversity index with treatment and sampling window and their interaction as explanatory variables, while controlling for sampling day (1 or 2) and sequencing depth. For all GAMs, sampling window was fitted with a cyclic cubic regression spline (bs=‘cc’), because of the cyclical nature of the 24-h sampling. Model fit was assessed using *gam.check*(). We visualized the model results using *plot_smooths*() from the ‘tidymv’ package (54).

*Microbiome beta diversity*: Unweighted and weighted UniFrac distances were calculated based on reads rarefied to the minimum read count (i.e. 546) using the *distance*() function from the ‘phyloseq’ package. Both distances take the phylogenetic distance between ASVs into account, but whereas weighted UniFrac considers reads as proxy for ASV abundance, unweighted UniFrac treats ASVs as either absent or present. Each distance matrix was analyzed using a permutational multivariate analysis of variance (PERMANOVA, permutations = 10 000) using the *adonis2*() function of the ‘vegan’ base package (Oksanen et al. 2022). Treatment and sampling window were set as main explanatory variables while controlling for sampling day. Treatment and sampling time points were placed in an interaction. To estimate treatment effects at each time point, a subsequent pairwise PERMANOVA was run employing the *pairwise.adonis2*() function of the ‘pairwiseAdonis’ package (Martinez Arbizu 2020). To identify 24-h cycles in beta diversity, we specified GAMs with PC1 values of each beta-diversity index as response and treatment and sampling window and their interaction as explanatory variables while controlling for sampling day and sequencing depth.

*Genus-level analyses*: We tested for genus-level oscillations and how these were affected by the delay in feeding. To identify which genera oscillated, we ran GAMs on the CLR-transformed abundances (Quinn et al. 2019) of the seven genera making up at least 1% of reads, using again treatment and sampling window and their interaction as explanatory variables, while controlling for sequencing depth and sampling day. Sampling window was fitted with a cyclic cubic regression spline, and models were quality checked and visualized as described before.

*pH differences and links to the microbiome*: To assess the changes in gut pH throughout the day, we fitted a GAM predicting pH, including treatment, sampling window and their interaction as well as sampling day as explanatory variables. To understand whether gut pH explained some variation around taxa showing diurnal fluctuations, we constructed a generalized linear model with the CLR-transformed abundance of the common core genera as response, and treatment group, pH, and their interaction as explanatory variables, while controlling for the sampling day.

## Results

### Gut microbial diversity and composition

**Figure 2.**
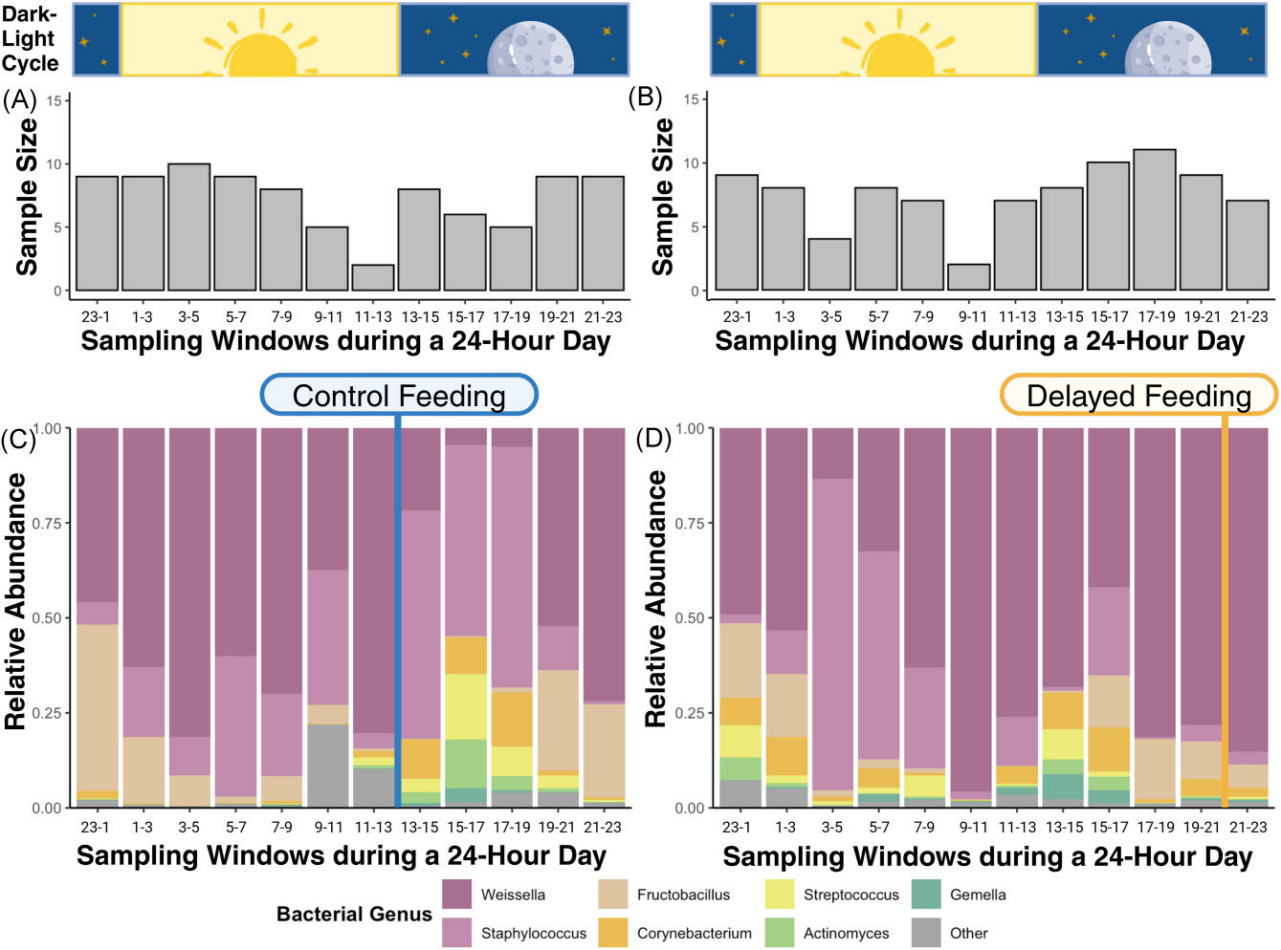
Number of samples collected after each sampling window and temporal variation in relative microbial abundances on the genera level. Shown are the results for the (A and C) control and (B and D) delayed-feeding groups. Feeding times are indicated for the control group with a blue and for the delayed-feeding group with a yellow vertical line. The unaltered dark–light cycle is depicted for reference. Bacterial genera making up <1% were grouped as ‘Other‘.

On average, only 12.0 (±6.4 SD) bacterial genera and 17.7 (±11.2 SD) unique ASVs were found in each of the 179 samples. In total, 698 ASVs were found among all 179 samples. ASVs of the bacterial class Bacilli (91.8%) and Actinobacteria (6.7%) made up >98% of all taxa (Fig. S5). *Weissella* was the dominant bacterial genus making up 52.3%, followed by *Staphylococcus* (20.9%), *Fructobacillus* (12.7%), *Corynebacterium* (5.0%), *Streptococcus* (3.1%), *Actinomyces* (1.5%), and *Gemella* (1.2%; Fig. 2, Fig. S6). In the control group, the relative abundance of *Corynebacterium* and *Actinomyces* peaked 2–6 h after the light was switched off and the food was provisioned (Fig. 2C). In contrast, *Corynebacterium* increased less steeply in the delayed-feeding group after the light was turned off, and peaked a second time 4–6 h after the delayed feeding (Fig. 2D). Oscillating patterns were also visible for other bacterial genera, such as *Weissella* and *Streptococcus*.

### Rhythms in gut microbial diversity and pH

**Figure 3.**
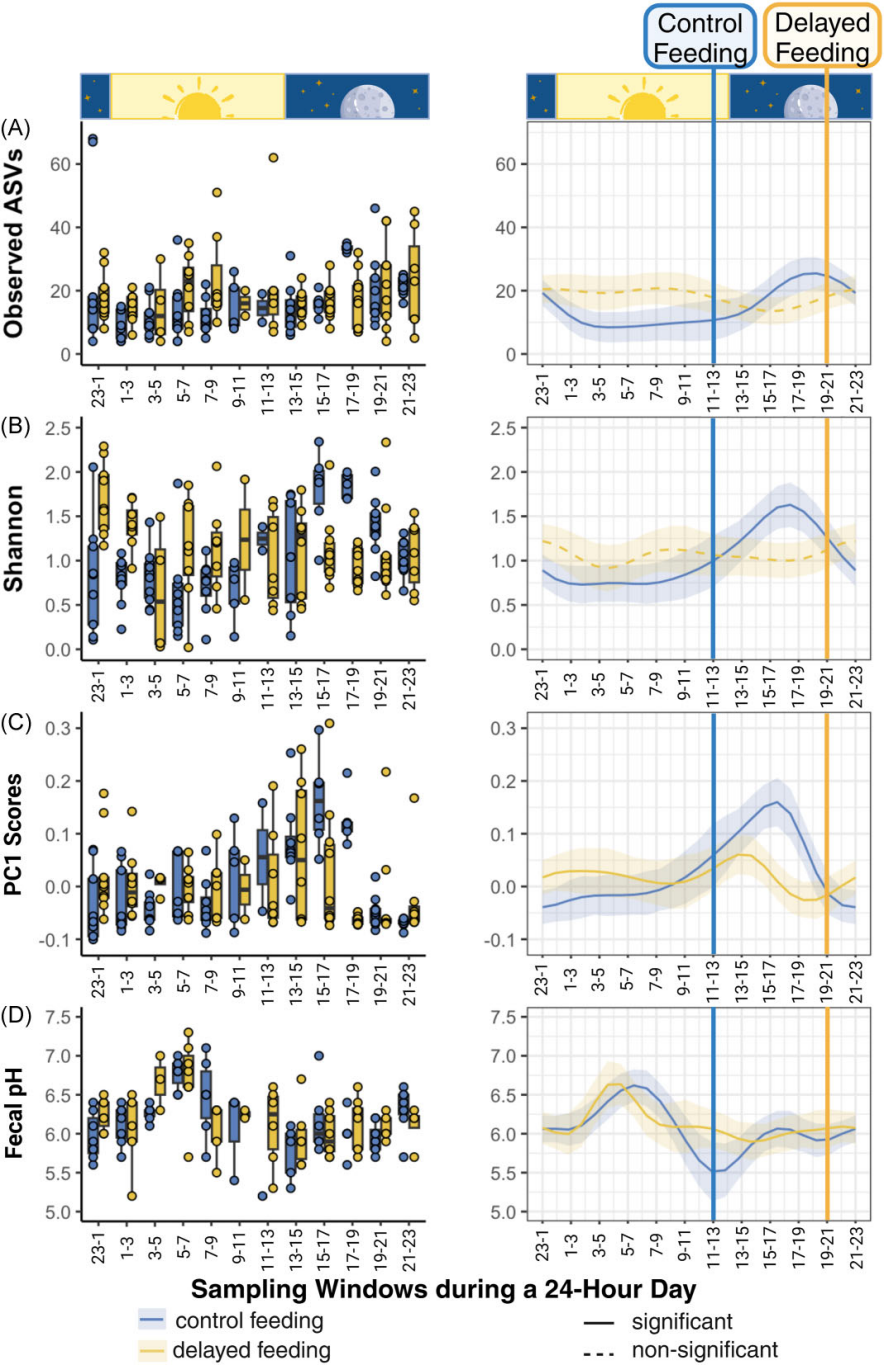
Diurnal variation in microbial alpha- and beta diversity, as well as faecal pH between the control (blue) and delayed (yellow)-feeding group. Alpha diversity was measured by (A) observed ASVs and (B) Shannon diversity; and (rarefied) beta diversity was measured by (C) PC1 score of unweighted UniFrac distances; (D) indicates faecal pH. All measures are shown as box plots (left panels) and model estimates (right panels). A solid line suggests a significant linear or nonlinear effect, whereas a dashed line indicates a nonsignificant effect of sampling window. The shaded area represents the 95% CI around the fitted line. The unaltered dark–light cycle is depicted for reference. Feeding times are indicated for the control group with a blue and for the delayed-feeding group with a yellow vertical line.

An 8-h delay in feeding disrupted rhythms in gut microbial alpha diversity, measured as observed ASV richness and Shannon index (Fig. 3A and B). While a sinus-shaped fluctuation in alpha diversity was apparent in the control group indicated by a significant nonlinear effect (observed ASV: effective degrees of freedom [edf] = 3.9, *F* = 3.93, *P* < .001; Shannon: edf = 4.3, *F* = 5.10, *P* < .001; Table S1, Fig. 3B), the oscillation was either weak (observed ASV: edf = 3.2, *F* = 0.88, *P* = .059) or not detectable (Shannon: edf = 4.2, *F* = 0.68, *P* = .249) in the delayed-feeding group.

The centroid of the weighted and unweighted UniFrac distances differed between sampling windows and this depended on the treatment group (weighted UniFrac: *R*^2^ = 0.15, *P* = .001; unweighted UniFrac: *R*^2^ = 0.10, *P* = .001; Fig. S7). Sampling day had a negligible effect on beta diversity (Table S2). Similar to alpha diversity, the PC1 scores for either beta-diversity index followed a nearly sinus-shaped oscillation in the control feeding group (unweighted UniFrac: edf = 2.8, *F* = 5.35, *P* < .001; weighted UniFrac: edf = 5.5, *F* = 8.39, *P* < .001; Fig. 3C; Table S3). While significantly nonlinear still, oscillations were shifted and disrupted in the delayed-feeding group (unweighted UniFrac: edf = 4.8, *F* = 1.48, *P* = .025; weighted UniFrac: edf = 4.9, *F* = 1.67, *P* = .015; Fig. 3C). The visualizations and the pairwise PERMANOVA results emphasized that this shift in centroid was particularly obvious in the times after the control group was fed and, then again, after the treatment group was fed (Table S4 and Fig. S7).

### Diurnally oscillating abundance at the genus level

**Figure 4.**
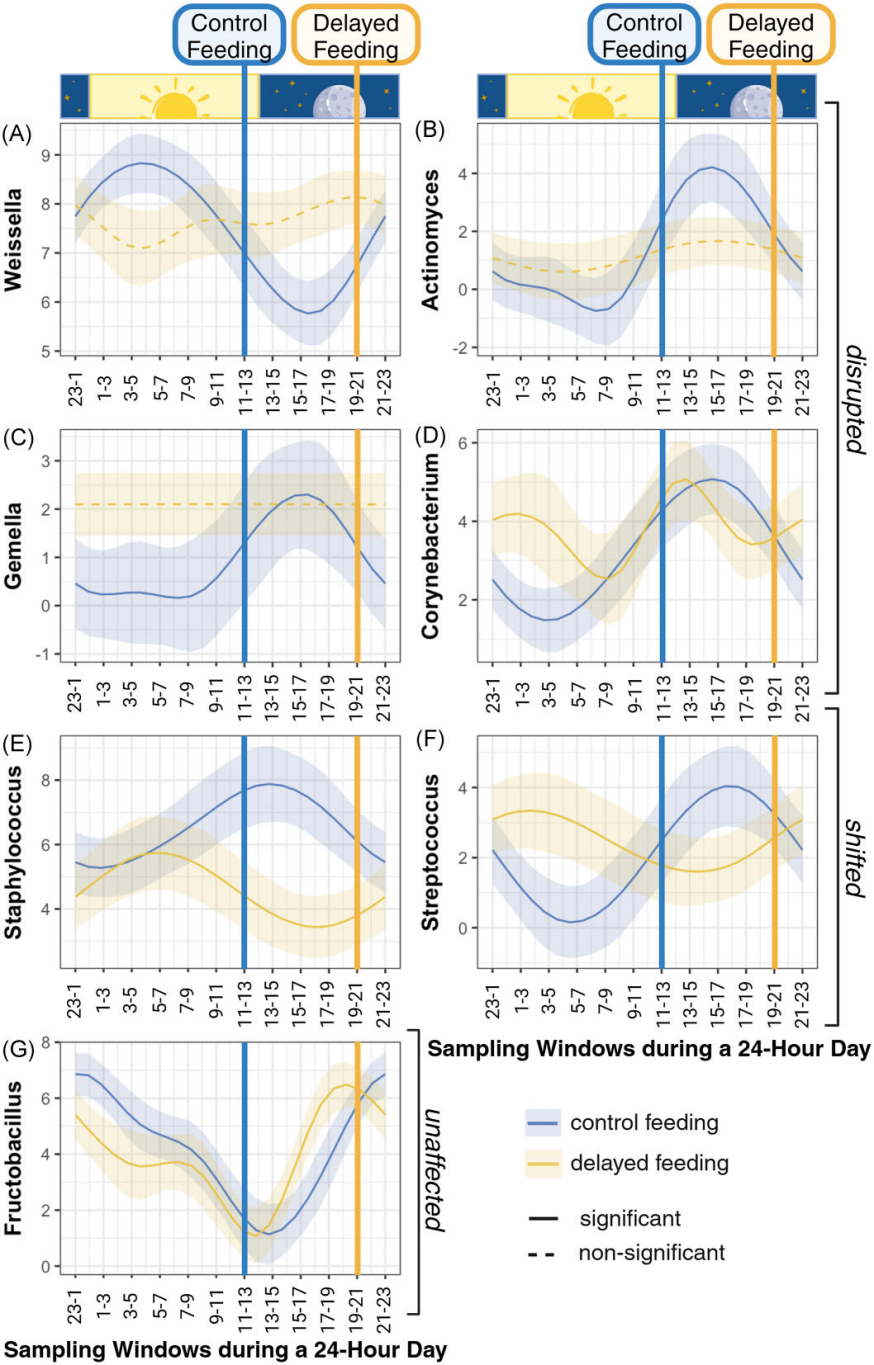
Bacterial genera oscillate over a 24-h time frame, but show differences between the control (blue) and delayed (yellow)-feeding group. A solid line suggests a significant linear or nonlinear effect, whereas a dashed line indicates a nonsignificant effect of sampling window. The shaded area represents the 95% CI around the fitted line. The unaltered dark–light cycle is depicted for reference. Feeding times are indicated for the control group with a blue and for the delayed-feeding group with a yellow vertical line.

Of the seven bacterial genera with >1% reads, all showed some form of nonlinear diurnal oscillation in relative abundance in the control group (Fig. 4, Table S5). Importantly though, in all but one genus (*Fructobacillus*; Fig. 4G), the oscillation in the delayed-feeding group was disrupted (Fig. 4A–D) or shifted (Fig. 4E and F): *Weissella*’s abundance, for example, peaked during the night period and then declined up until ~4 h after feeding in the control feeding group (edf = 2.8, *F* = 12.35, *P* < .001; Fig. 4A). In the delayed-feeding group, this oscillation was nonsignificant (edf = 3.0, *F* = 1.33, *P* = .130). In the case of *Corynebacterium*, the delay caused a highly irregular oscillation (control: edf = 2.4, *F* = 8.77, *P* < .001; treatment: edf = 3.6, *F* = 1.20, *P* = .021; Fig. 4D). The fluctuation of *Streptococcus* was nearly opposite to that found for *Weissella* in the control group (edf = 2.2, *F* = 6.25, *P* < .001) but, while delayed, remained nonlinear in the treatment group (edf = 1.8, *F* = 1.59, *P* = .020; Fig. 4F), which suggests a phase shift in line with feeding times. Single effects of treatment, sampling day, and sequencing depth were rare (Table S5).

### Faecal pH in relation to microbial diversity and abundance

**Figure 5.**
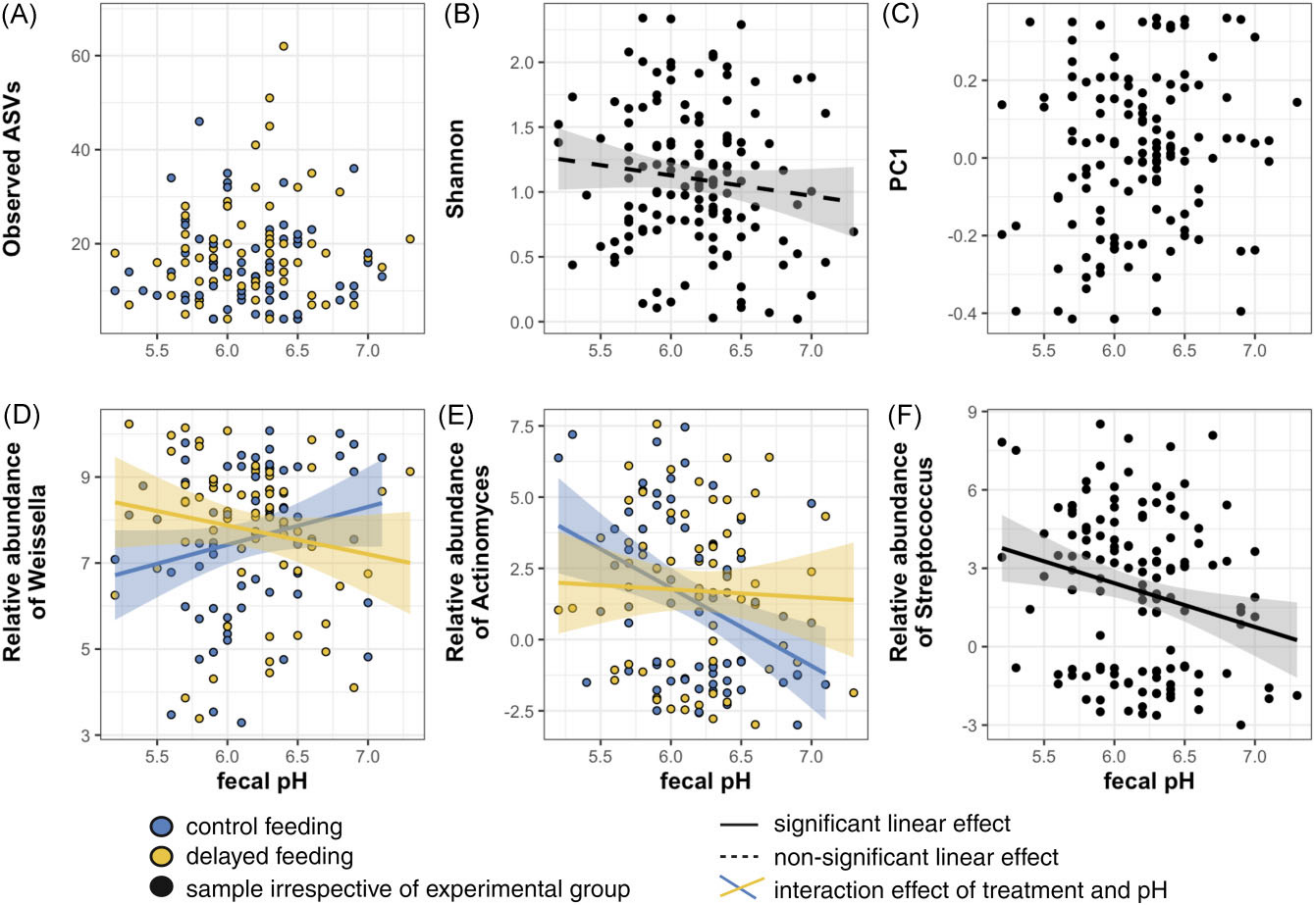
Relationship between faecal pH, bacterial diversity (i.e. observed ASVs, Shannon, and unweighted UniFrac distances) and the (CLR-transformed) relative abundance of certain genera. Samples from the control and delayed-feeding group were coloured in blue and yellow, respectively. Significant relationships were drawn as solid lines and trends as dashed lines.

Faecal pH oscillated nonlinearly over the 24-h period without significant differences between the control and delayed-feeding group (edf = 3.9, *F* = 7.56, *P* < .001; edf = 6.5, *F* = 2.83, *P* = .002; Fig. 3D, Table S6). Assessing whether faecal pH predicted microbial diversity or the abundance certain genera uncovered few effects (Fig. 5, Table S7): Only the Shannon diversity index tended weakly to decline at higher pH (estimate: −0.31, *P* = .050). The abundance of *Weissella* declined at low faecal pH in the control feeding group, while increasing in the delayed-feeding group (pH × treatment interaction: estimate: −1.65, *P* = .019; Fig. 5D). Similarly, *Actinomyces* showed an interaction effect (estimate: 2.39, *P* = .042; Fig. 5E). *Streptococcus* increased in abundance in more acidic conditions and this did not depend on the treatment group (estimate: −2.67, *P* = .002; Fig. 5F).

## Discussion

Gut microbial rhythms are crucial for host physiology and function (Frazier and Chang 2020, Segers and Depoortere 2021), yet we have a poor understanding of the cues that prompt diurnal fluctuations in gut bacteria (Schmid et al. 2023). Here, we demonstrate experimental evidence that gut microbial rhythms exist in a nonmodel bat species, and that these rhythms are synchronized, at least in part, by feeding. Delaying the feeding cue shifted peaks in alpha diversity and beta diversity, and disrupted or shifted the circadian fluctuation of some common gut commensals with functionally important roles in metabolizing nutrients. In contrast, oscillations in faecal pH were largely unaffected by a delay in feeding time and were only weakly associated with changes to microbiota composition.

Our experiment found clear diurnal gut microbial rhythms in the control feeding group in the form of fluctuations in gut microbial diversity, changes in gut microbial composition, and oscillations in common gut microbial commensals. Although the bacterial richness of our captive population was an order of magnitude lower than that found in wild *L. yerbabuenae* (Gaona et al. 2020, Víquez-R et al. 2021), the major bacterial families remain represented (Fountain et al. 2022). We speculate that the reduced richness is because of their simple and standardized diet over decades in captivity compared with the up to 34 plant species visited by the species in nature (Tremlett et al. 2024). And while the low diversity may not fully capture the dynamics of more complex microbiomes found in wild *L. yerbabuenae* (Carrillo-Araujo et al. 2015, Víquez-R et al. 2021), this does not negate that feeding time affected diurnal gut microbial dynamics. Unlike previous experimental work, we were able to dissociate feeding from light cues by delaying feeding in one group. The delay in feeding resulted in two peaks or disrupted rhythmicity within the same 24-h period.

Among the core bacterial genera in the control group of captive *L. yerbabuenae*, diurnal rhythmicity seemed to be the rule rather than the exception. *Weissella* declined in abundance at the start of the dark cycle, which also coincided with feeding, whereas other *Bacillota* (formerly known as *Firmicutes*) and *Actinomycetota* (formerly known as *Actinobacteria*) increased. *Bacillota* were also considered oscillators in laboratory mice (Brooks et al. 2021) and clown fish (Parris et al. 2019). In wild meerkats too, the most common gut commensal *Clostridium* belongs to the phylum *Bacillota* and reached its highest abundance in the morning during feeding bouts and declined thereafter (Risely et al. 2021). *Bacillota* also reach up to 66% of all gut bacteria in wild *L. yerbabuenae* (Gaona et al. 2019, 2020, Víquez-R et al. 2021), where they likely aid the host in processing sugars (Ingala et al. 2021), and synthesizing short chain fatty acids (Kolmeder et al. 2012, Youngblut et al. 2019). *Streptococcus* and *Weissella*, both lactic acid-producing bacteria, are also enriched during the winter period, when wild female *L. yerbabuenae* migrate to the Sonoran Desert in Mexico, and exclusively feed on the nectar from a few flowering columnar cacti species (Sperr et al. 2011, Víquez-R et al. 2021). We hypothesize, therefore, that the diurnal fluctuations of these bacterial genera may allow the host to capitalize quickly on the few nutrients available from its sugary diet.

Evidence for diurnal fluctuations of bacteria involved in food assimilation could underscore their ecological and evolutionary importance because the fluctuations match peak feeding times when the microbiome-mediated metabolic support is most needed. However, this is an inference that will need to be tested with assays that, besides their taxonomy, can map the functional (i.e. metagenomics) and realized (i.e. multiomics) niche of gut bacteria (Worsley et al. 2024b). Moreover, because we did not quantify the food-associated microbiome, the extent to which the observed microbial fluctuations reflect transient, microbiome dynamics is unclear. In humans, ~15% of the microbiome is associated with transient, food-borne microbes (Lee et al. 2024). The simple digestive tract evolved as adaptation to flight to enhance paracellular nutrient absorption (Caviedes-Vidal et al. 2007), and rapid gut transit times led others to suggest that bats may be less dependent on gut symbionts than other mammals and feature more transient bacteria in their faecal microbiome (Song et al. 2020, Jones et al. 2022, Williams and Fontaine 2024). However, this remains to be shown (Hird 2020). On the other hand, bats’ expansive intestinal villus lining in the gut epithelium could equally augment the cross-talk between microbiota and epithelial cells and boost nutrient uptake when gut transit times are rapid (Price et al. 2015). Even if transient bacteria were common in bats, transients contribute to the microbiome function and dysregulation (Lee et al. 2024), suggesting that their diurnal oscillations are still functionally relevant.

We also found that the diurnal fluctuation of gut pH was unaffected by a delay in feeding. This was somewhat surprising, because in cow rumen the pH decreased after feeding as lactic acid-producing bacteria (phylum: *Bacillota*) multiply (Shaani et al. 2018). A pH drop could have functional benefits because a low pH improves the synthesis and assimilation of short-chain fatty acids (Aschenbach et al. 2011, Blaak et al. 2020). Since we considered the abiotic gut environment paramount in shaping gut microbial diversity, we expected a strong relationship between gut pH and members of the bacterial community. And yet, only *Streptococcus* decreased with pH, irrespective of treatment. Lactic acid-producing bacteria taxa, such as *Streptococcus*, may themselves lower gut pH, and, consequently, engineer the niche ‘gut’ for the rest of the microbial community (Firrman et al. 2022). Future studies may want to explore whether ecological interactions between co-occurring members of the gut microbial community, rather than the abiotic environment of the gut *per se*, cosynchronize gut microbial dynamics. Such fine-scale community dynamics might best be addressed with a sampling scheme that can differentiate between individuals to truly capture longitudinal dynamics and stability of the microbiota within individuals (Marsh et al. 2024).

Overall, we show that delaying feeding alters within host microbial dynamics in a nonmodel organism. Ignoring diurnal microbial dynamics will muddle our understanding of how host-mediated processes (e.g. energy assimilation and immune responses) aligned with microbial rhythms (Gillingham et al. 2024), and thus misrepresent the role of gut bacteria in host ecology and fitness (Allaband et al. 2024). Specifically, disruptions may impact host health. For instance, disrupted microbial rhythms were found to increase susceptibility to *Salmonella typhimurium* in laboratory mice (Brooks et al. 2021), and humans with unstable host–microbe interactions had increased risk of metabolic disease (Reitmeier et al. 2020) and mental disorders (Teichman et al. 2020). Disturbances for wildlife that impact foraging behaviour (such as artificial light at night in bats; e.g. Stone et al. 2015, Seewagen et al. 2023, Stidsholt et al. 2024) may therefore also dysregulate gut microbial rhythms with possible consequences for host health.

## Supplementary Material

fiaf012_Supplemental_File

## Data Availability

R code and data are accessible from https://github.com/DominikWSchmid/Lepto_circadianrythm.
