# Supplementary material for: Delayed feeding disrupts diurnal oscillations in the gut microbiome of a neotropical bat in captivity
Source: FEMS Microbiol Ecol. 2025 Jan 22;101(2):fiaf012. doi: 10.1093/femsec/fiaf012 (PMC11783575; doi:10.1093/femsec/fiaf012)
Supplement: fiaf012_Supplemental_File [file fiaf012_supplemental_file.docx]

**Supplementary Figure and Tables**


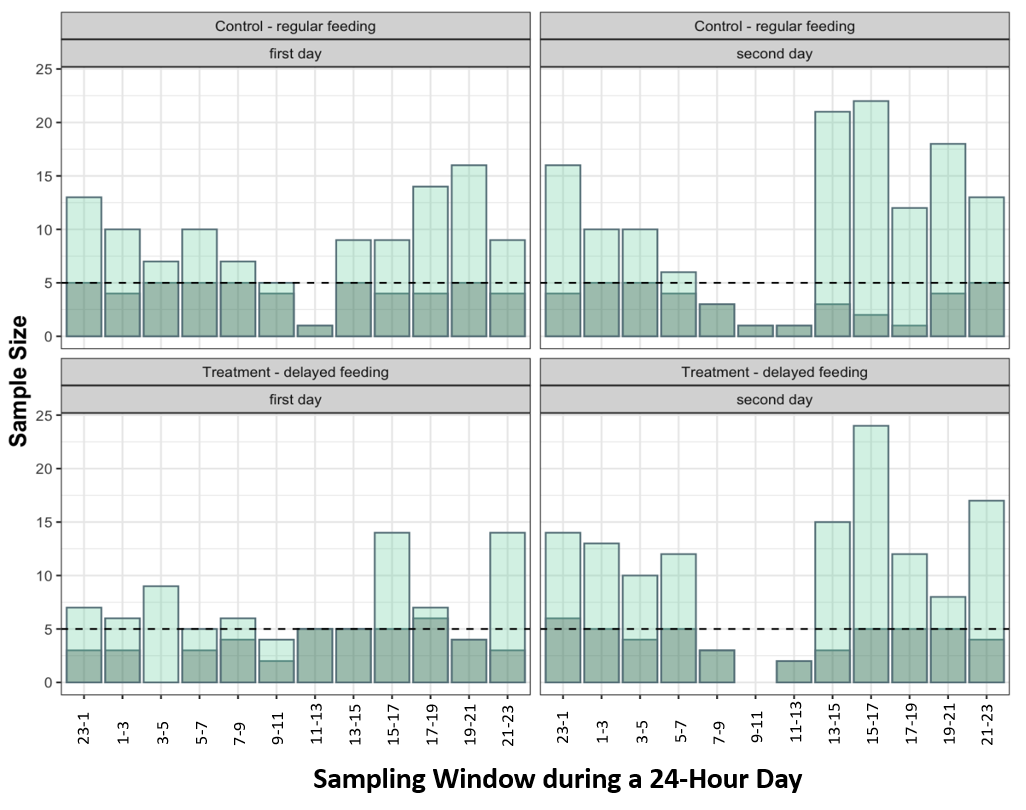


**Supplementary Figure 1. Number of fecal samples collected and sequenced from each sampling window.** The graph is divided by collection day and control or delayed feeding group. Light green bars show number of fecal samples collected, dark green bars indicate the number of samples retained after filtering and quality assessment (n=179). The dashed line indicates the intended sample size.

**
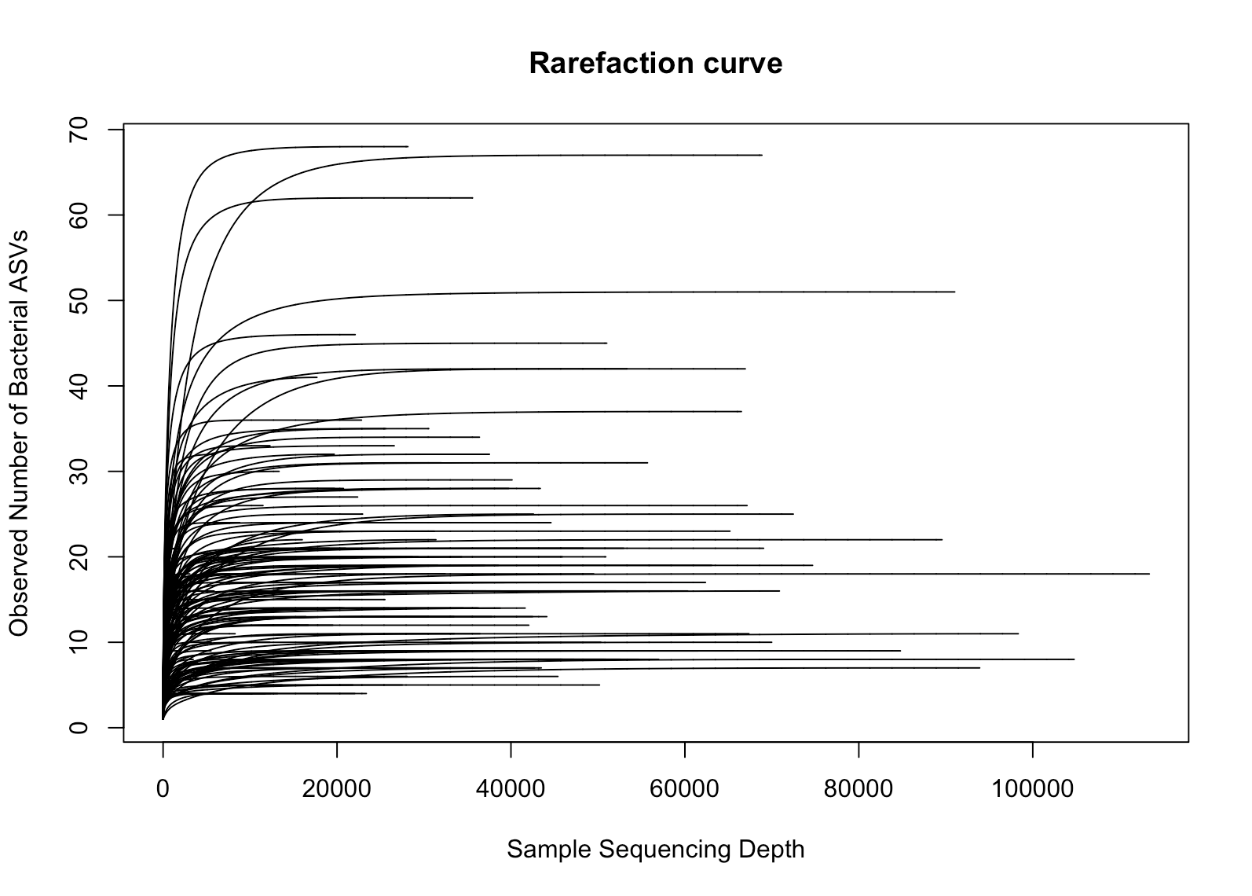
**

**Supplementary Figure 2. Rarefaction curve for 179 samples post filtering and decontamination workflow.**

**
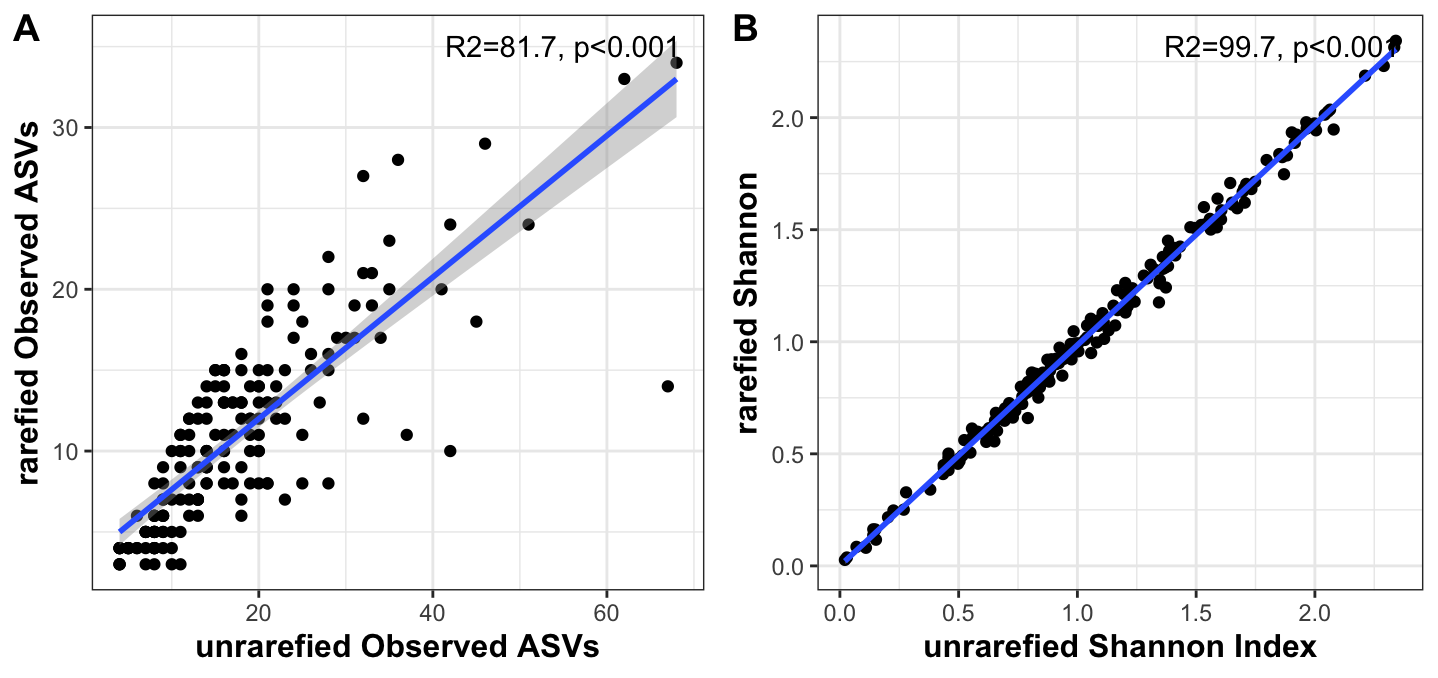
**

**Supplementary Figure 3. Correlation between rarefied vs. unrarefied A) Observed ASVs and B) Shannon index.** Minimum read counts (i.e., 546) was used for rarefying.

**
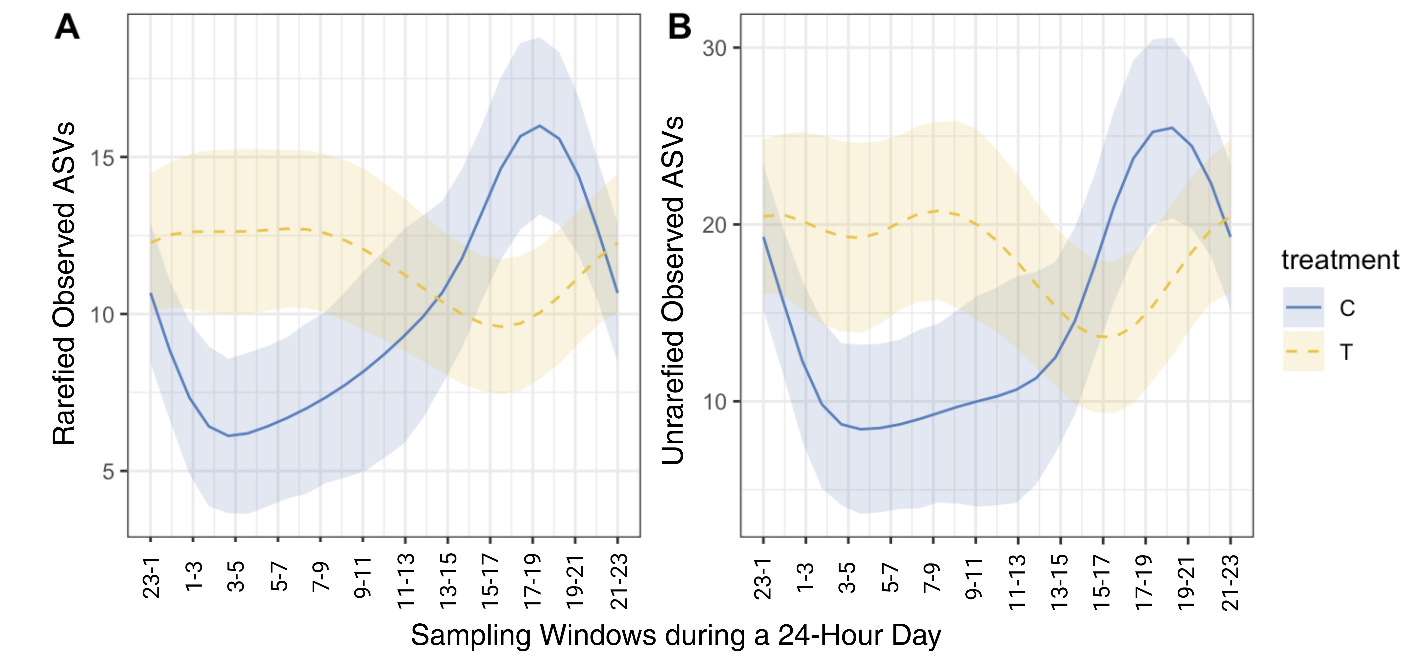
**

**Supplementary Figure 4. Visualization of the comparison between A) rarefied and B) unrarefied GAM results for Observed ASVs.** The lines represent the predicted values of the GAM model and the shaded area the 95% CI.

**
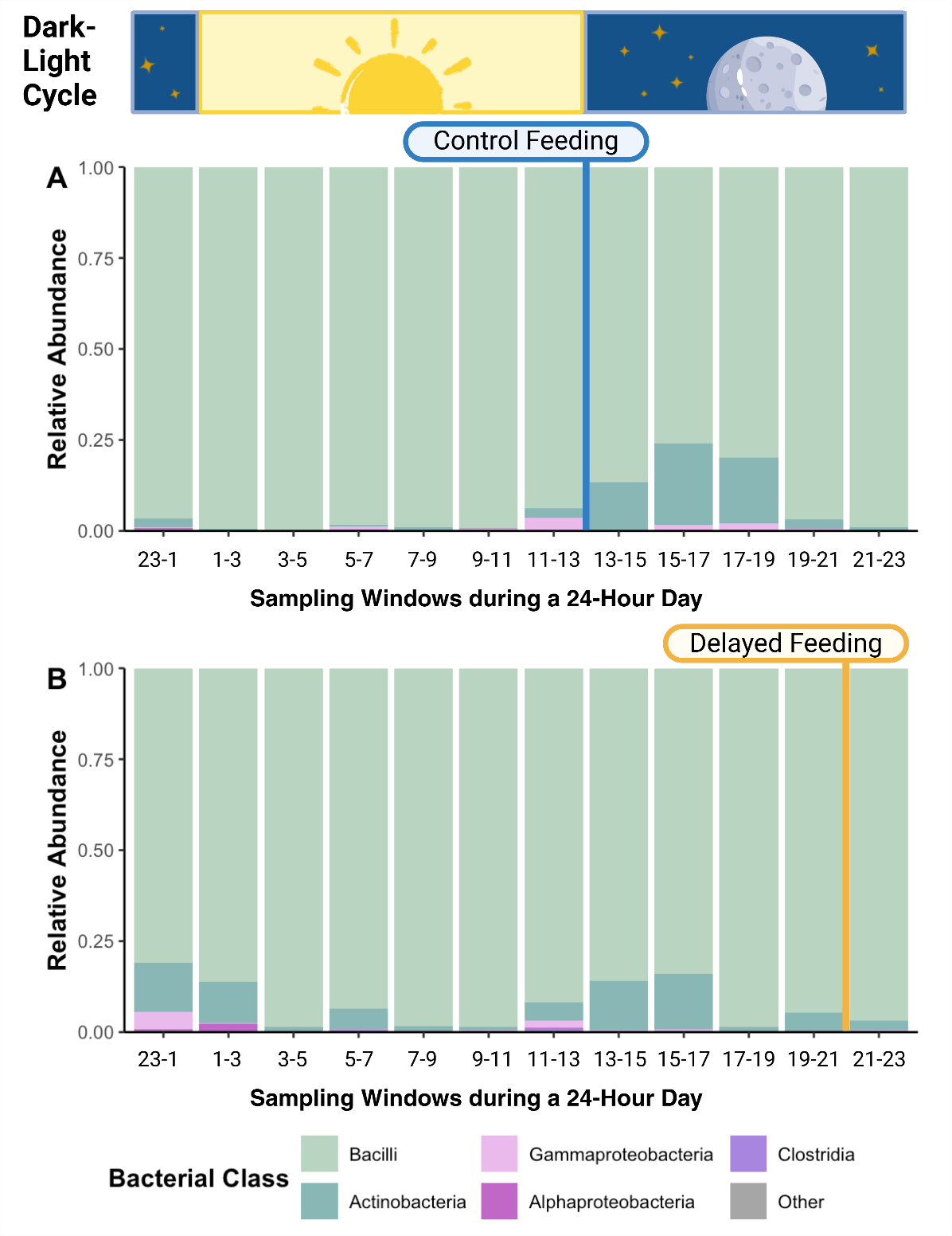
**

**Supplementary Figure 5. Temporal variation in relative microbial abundances coloured by bacterial classes for samples taken from A) the control feeding group and B) the delayed feeding group.** Feeding times are indicated for the control group with a blue vertical line and for the delayed feeding group with a yellow vertical line. The unaltered dark-light cycle is depicted for reference. Bacterial classes making up less than 0.1% were grouped as ‘Other’.

**
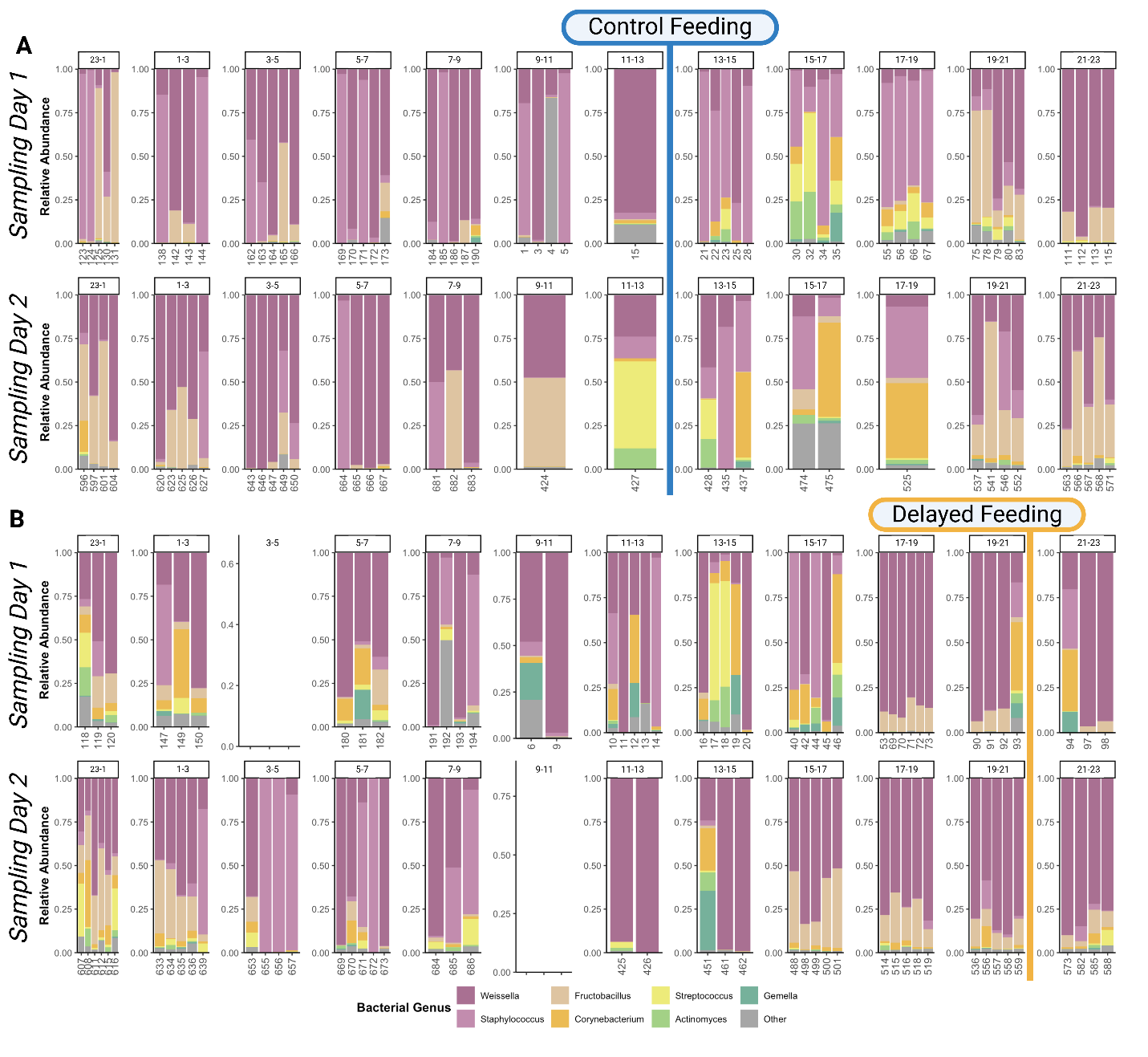
**

**Supplementary Figure 6. Relative microbial abundances coloured by bacterial genera for each sample taken from A) the control feeding group and B) the delayed feeding group, and separated by sampling window and sampling day.** Bacterial genera making up less than 1% overall were grouped as ‘Other’. Sample IDs given on the x-axis.

**Supplementary Table 1. Summary results for the generalized additive models looking at the effect of treatment, sampling window, sampling day and sequencing depth on unrarefied and rarefied alpha diversity indices. Displayed are the results for a) Observed ASVs and b) Shannon diversity.** edf=effective degree of freedom; ref.df=reference degree of freedom; Significant results are presented in bold; n=179.

| ***a) Observed ASVs (unrarefied)*** |  |  |  |  |
| --- | --- | --- | --- | --- |
| **Smooth Terms** | **edf** | **ref.df** | **statistic** | **p-value** |
| s(sampling window):Control | 3.94 | 8.00 | 3.93 | **<0.001** |
| s(sampling window):Delayed | 3.16 | 8.00 | 0.88 | 0.059 |
| s(sequencing depth) | 4.02 | 4.96 | 5.22 | **<0.001** |
| **Fixed Term** | **estimate** | **std.error** | **statistic** | **p-value** |
| Intercept | 15.54 | 1.25 | 12.41 | **<0.001** |
| Treatment | 2.78 | 1.55 | 1.79 | 0.075 |
| Sampling day | 2.69 | 1.59 | 1.70 | 0.091 |
|  |  |  |  |  |
| ***Observed ASVs (rarefied)*** |  |  |  |  |
| **Smooth Terms** | **edf** | **ref.df** | **statistic** | **p-value** |
| s(sampling window):Control | 3.83 | 8.00 | 3.86 | **<0.001** |
| s(sampling window):Delayed | 2.40 | 8.00 | 0.62 | 0.088 |
| s(sequencing depth) | 2.91 | 3.62 | 0.98 | 0.351 |
| **Fixed Term** | **estimate** | **std.error** | **statistic** | **p-value** |
| Intercept | 10.32 | 0.68 | 15.13 | **<0.001** |
| Treatment | 1.27 | 0.84 | 1.54 | 0.132 |
| Sampling day | 0.77 | 0.86 | 0.90 | 0.369 |
|  |  |  |  |  |
| ***b) Shannon (unrarefied)*** |  |  |  |  |
| **Smooth Terms** | **edf** | **ref.df** | **statistic** | **p-value** |
| s(sampling window):Control | 4.31 | 8.00 | 5.10 | **<0.001** |
| s(sampling window):Delayed | 4.17 | 8.00 | 0.68 | 0.249 |
| s(sequencing depth) | 1.00 | 1.00 | 18.06 | **<0.001** |
| **Fixed Term** | **estimate** | **std.error** | **statistic** | **p-value** |
| Intercept | 1.03 | 0.05 | 19.06 | **<0.001** |
| Treatment | 0.05 | 0.07 | 0.68 | 0.499 |
| Sampling day | 0.10 | 0.07 | 1.49 | 0.136 |
|  |  |  |  |  |
| ***b) Shannon (rarefied)*** |  |  |  |  |
| **Smooth Terms** | **edf** | **ref.df** | **statistic** | **p-value** |
| s(sampling window):Control | 4.28 | 8.00 | 5.15 | **<0.001** |
| s(sampling window):Delayed | 3.69 | 8.00 | 0.52 | 0.333 |
| s(sequencing depth) | 1.00 | 1.00 | 18.36 | **<0.001** |
| **Fixed Term** | **estimate** | **std.error** | **statistic** | **p-value** |
| Intercept | 1.01 | 0.05 | 18.96 | **<0.001** |
| Treatment | 0.04 | 0.07 | 0.68 | 0.495 |
| Sampling day | 0.11 | 0.07 | 1.61 | 0.109 |

**
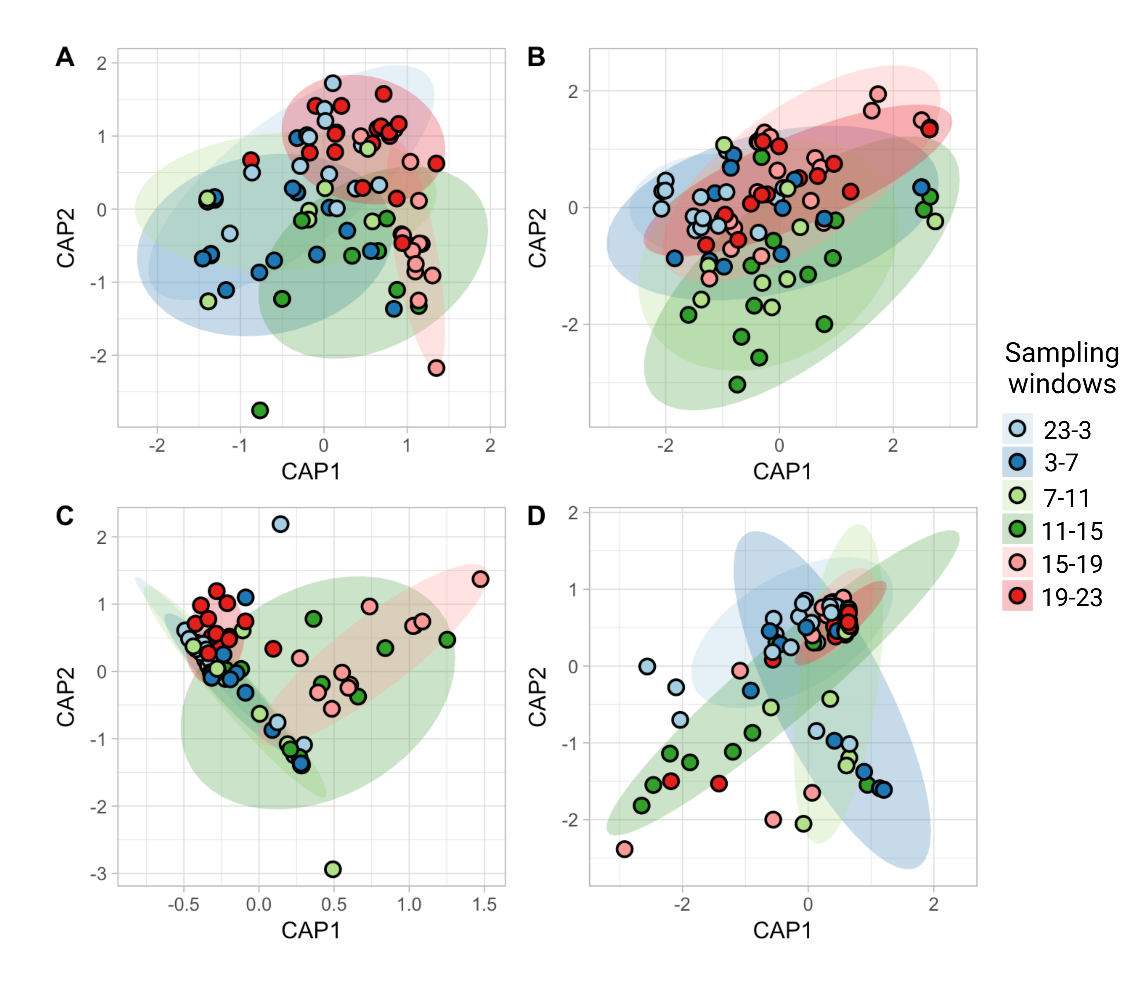
**

**Supplementary Figure 7. Constrained ordination of beta-diversity computed from A-B) unweighted and C-D) weighted Unifrac distances and comparing the A+C) control and B+D) the delayed feeding group.** Points are colored by sampling window, which were grouped for visualization purposes.

**Supplementary Table 2. Permanova results comparing differences in rarefied A) unweighted Unifrac and B) weighted Unifrac between the control and delayed feeding group for each sampling window, sampling day and sequencing depth.** Significant results are bold; n=179.

| ***a) Unweighted Unifrac*** | **R2** | **F-statistic** | **p-value** |
| --- | --- | --- | --- |
| Sampling day | 0.01 | 1.92 | 0.074 |
| Sequencing depth | 0.01 | 1.23 | 0.256 |
| Treatment*sampling window | 0.10 | 1.84 | **0.001** |
|  |  |  |  |
| ***b) Weighted Unifrac*** | **R2** | **F-statistic** | **p-value** |
| Sampling day | 0.02 | 3.69 | **0.025** |
| Sequencing depth | 0.01 | 2.13 | 0.106 |
| Treatment*sampling window | 0.15 | 3.33 | **0.001** |

**Supplementary Table 3. Summary results for the generalized additive models looking at the effect of treatment, sampling window, sampling day and sequencing depth on rarefied beta diversity indices. Displayed are the results for a) Unweighted Unifrac and b) Weighted Unifrac distances.** edf=effective degree of freedom; ref.df=reference degree of freedom; Significant results are presented in bold; n=179.

| ***a) Unweighted Unifrac*** |  |  |  |  |
| --- | --- | --- | --- | --- |
| **Smooth Terms** | **edf** | **ref.df** | **statistic** | **p.value** |
| s(sampling window):Control | 2.77 | 8.00 | 5.35 | **<0.001** |
| s(sampling window):Delayed | 4.76 | 8.00 | 1.48 | **0.025** |
| s(sequencing depth) | 1.00 | 1 | 0.70 | 0.403 |
| **Fixed Term** | **estimate** | **std.error** | **statistic** | **p.value** |
| Intercept | 0.03 | 0.02 | -1.14 | 0.258 |
| Treatment | 0.03 | 0.03 | 0.93 | 0.353 |
| Sampling day | 0.01 | 0.03 | 0.49 | 0.619 |
|  |  |  |  |  |
| ***b) Weighted Unifrac*** |  |  |  |  |
| **Smooth Terms** | **edf** | **ref.df** | **statistic** | **p.value** |
| s(sampling window):Control | 5.52 | 8.00 | 8.39 | **<0.001** |
| s(sampling window):Delayed | 4.94 | 8.00 | 1.67 | **0.015** |
| s(sequencing depth) | 1.00 | 1 | 0.47 | 0.491 |
| **Fixed Term** | **estimate** | **std.error** | **statistic** | **p.value** |
| Intercept | 0.02 | 0.01 | 2.35 | **0.020** |
| Treatment | -0.01 | 0.01 | -0.46 | 0.576 |
| Sampling day | -0.03 | 0.01 | -2.52 | **0.013** |

**Supplementary Table 4. Pairwise Permanova results comparing differences in A) microbial community composition (unweighted Unifrac) and B) structure (weighted Unifrac) between the control and delayed feeding group for each sampling window.** Significant results are bold; n=179.

| **A) Unweighted Unifrac distances** | | |  |  |
| --- | --- | --- | --- | --- |
| *Sampling window + comparison* | | *df* | *F-statistic* | *p-value* |
| 23-1 Control - Treatment | | 1,17 | 2.40 | **0.007** |
| 1-3 Control - Treatment | | 1,16 | 5.14 | **0.004** |
| 3-5 Control - Treatment | | 1,13 | 1.53 | 0.141 |
| 5-7 Control - Treatment | | 1,16 | 2.66 | **0.027** |
| 7-9 Control - Treatment | | 1,14 | 1.47 | 0.162 |
| 9-11 Control - Treatment | | 1,6 | 0.71 | 0.675 |
| 11-13 Control - Treatment | | 1,8 | 0.49 | 0.999 |
| 13-15 Control - Treatment | | 1,15 | 0.77 | 0.596 |
| 1517 Control - Treatment | | 1,15 | 2.12 | 0.053 |
| 17-19 Control - Treatment | | 1,15 | 6.17 | **0.003** |
| 19-21 Control - Treatment | | 1,17 | 2.35 | 0.029 |
| 21-23 Control - Treatment | | 1,15 | 0.87 | 0.540 |
|  |  |  |  |  |
| **B) Weighted Unifrac distances** | | |  |  |
| *sampling window + comparison* | | *Df* | *F-statistic* | *p-value* |
| 23-1 Control - Treatment | | 1,17 | 4.30 | **0.006** |
| 1-3 Control - Treatment | | 1,16 | 3.04 | **0.031** |
| 3-5 Control - Treatment | | 1,13 | 7.76 | **0.012** |
| 5-7 Control - Treatment | | 1,16 | 1.35 | 0.298 |
| 7-9 Control - Treatment | | 1,14 | 1.90 | 0.177 |
| 9-11 Control - Treatment | | 1,6 | 0.63 | 0.597 |
| 11-13 Control - Treatment | | 1,8 | 1.05 | 0.273 |
| 13-15 Control - Treatment | | 1,15 | 2.10 | 0.118 |
| 1517 Control - Treatment | | 1,15 | 3.68 | **0.045** |
| 17-19 Control - Treatment | | 1,15 | 58.1 | **0.002** |
| 19-21 Control - Treatment | | 1,17 | 1.37 | 0.279 |
| 21-23 Control - Treatment | | 1,15 | 2.65 | **0.001** |

**Supplementary Table 5. Summary results for the generalized additive model looking at the effect of treatment, sampling window, and sequencing depth on the (clr-transformed) abundance of the seven bacterial genera with more than 1% reads.** edf=effective degree of freedom; ref.df=reference degree of freedom; Significant results are presented in bold; n=179.

| ***a) Weissella*** |  |  |  |  |
| --- | --- | --- | --- | --- |
| **Smooth Terms** | **edf** | **ref.df** | **statistic** | **p.value** |
| s(sampling window):Control | 2.76 | 4.00 | 12.35 | **<0.001** |
| s(sampling window):Delayed | 2.95 | 4.00 | 1.33 | 0.130 |
| s(sequencing depth) | 3.17 | 4.00 | 1.67 | 0.098 |
| **Fixed Term** | **estimate** | **std.error** | **statistic** | **p.value** |
| Intercept | 7.44 | 0.18 | 42.29 | **<0.001** |
| Treatment | 0.25 | 0.22 | 1.67 | 0.245 |
| Sampling day | 0.37 | 0.22 | 1.66 | 0.098 |
|  |  |  |  |  |
| ***b) Staphylococcus*** |  |  |  |  |
| **Smooth Terms** | **edf** | **ref.df** | **statistic** | **p.value** |
| s(sampling window):Control | 2.01 | 4.00 | 3.06 | **<0.001** |
| s(sampling window):Delayed | 1.76 | 4.00 | 2.52 | **0.002** |
| s(sequencing depth) | 2.04 | 2.57 | 1.11 | 0.248 |
| **Fixed Term** | **estimate** | **std.error** | **statistic** | **p.value** |
| Intercept | 6.36 | 0.33 | 19.12 | **<0.001** |
| Treatment | -1.84 | 0.41 | -4.51 | **<0.001** |
| Sampling day | -0.54 | 0.42 | -1.30 | 0.195 |
|  |  |  |  |  |
| ***c) Fructobacillus*** |  |  |  |  |
| **Smooth Terms** | **edf** | **ref.df** | **statistic** | **p.value** |
| s(sampling window):Control | 3.59 | 4.00 | 21.27 | **<0.001** |
| s(sampling window):Delayed | 3.76 | 4.00 | 16.53 | **<0.001** |
| s(sequencing depth) | 2.96 | 3.71 | 4.19 | **0.009** |
| **Fixed Term** | **estimate** | **std.error** | **statistic** | **p.value** |
| Intercept | 4.54 | 0.24 | 18.91 | **<0.001** |
| Treatment | -0.29 | 0.30 | -0.99 | 0.323 |
| Sampling day | 1.27 | 0.30 | 4.19 | **<0.001** |
|  |  |  |  |  |
| ***d) Corynebacterium*** | |  |  |  |
| **Smooth Terms** | **edf** | **ref.df** | **statistic** | **p.value** |
| s(sampling window):Control | 2.36 | 4.00 | 8.77 | **<0.001** |
| s(sampling window):Delayed | 3.57 | 4.00 | 2.67 | **0.021** |
| s(sequencing depth) | 1.00 | 1.00 | 7.12 | **0.009** |
| **Fixed Term** | **estimate** | **std.error** | **statistic** | **p.value** |
| Intercept | 3.15 | 0.26 | 12.23 | **<0.001** |
| Treatment | 0.67 | 0.32 | 2.11 | **0.037** |
| Sampling day | 0.59 | 0.32 | 1.84 | 0.068 |
|  |  |  |  |  |
| ***e) Streptococcus*** |  |  |  |  |
| **Smooth Terms** | **edf** | **ref.df** | **statistic** | **p.value** |
| s(sampling window):Control | 2.22 | 4.00 | 6.25 | **<0.001** |
| s(sampling window):Delayed | 1.78 | 4.00 | 1.59 | **0.020** |
| s(sequencing depth) | 1.00 | 1.00 | 0.01 | 0.950 |
| **Fixed Term** | **Estimate** | **std.error** | **statistic** | **p.value** |
| Intercept | 2.14 | 0.33 | 6.54 | **<0.001** |
| Treatment | 0.41 | 0.0 | 1.02 | 0.308 |
| Sampling day | -0.20 | 0.41 | -0.43 | 0.661 |
|  |  |  |  |  |
| ***f) Actinomyces*** |  |  |  |  |
| **Smooth Terms** | **edf** | **ref.df** | **statistic** | **p.value** |
| s(sampling window):Control | 3.30 | 4.00 | 9.66 | **<0.001** |
| s(sampling window):Delayed | 1.28 | 4.00 | 0.83 | 0.066 |
| s(sequencing depth) | 1.00 | 1.00 | 5.98 | 0.016 |
| **Fixed Term** | **estimate** | **std.error** | **statistic** | **p.value** |
| Intercept | 1.37 | 0.31 | 4.43 | **<0.001** |
| Treatment | -0.23 | 0.38 | -0.62 | 0.538 |
| Sampling day | 0.54 | 0.39 | 1.39 | 0.165 |
|  |  |  |  |  |
| ***g) Gemella*** |  |  |  |  |
| **Smooth Terms** | **edf** | **ref.df** | **statistic** | **p.value** |
| s(sampling window):Control | 2.66 | 4.00 | 2.32 | **0.014** |
| s(sampling window):Delayed | 0.01 | 4.00 | 0.01 | 0.366 |
| s(sequencing depth) | 7.28 | 8.12 | -1.14 | 0.363 |
| **Fixed Term** | **Estimate** | **std.error** | **statistic** | **p.value** |
| Intercept | 0.94 | 0.31 | 3.06 | **0.003** |
| Treatment | 1.16 | 0.38 | 3.07 | **0.003** |
| Sampling day | -1.02 | 0.39 | -2.63 | **0.009** |

**Supplementary Table 6. Summary results for the generalized additive model looking at the effect of treatment, sampling window, and sampling day on fecal pH.** edf=effective degree of freedom; ref.df=reference degree of freedom; Significant results are presented in bold; n=134.

| ***Fecal pH*** |  |  |  |  |
| --- | --- | --- | --- | --- |
| **Smooth Terms** | **edf** | **ref.df** | **statistic** | **p.value** |
| s(sampling window):Control | 3.88 | 8.00 | 5.11 | **<0.001** |
| s(sampling window):Delayed | 6.52 | 8.00 | 2.83 | **0.002** |
| **Fixed Term** | **estimate** | **std.error** | **statistic** | **p.value** |
| Intercept | 6.08 | 0.05 | 124.01 | **<0.001** |
| Treatment | 0.03 | 0.06 | 0.56 | 0.578 |
| Sampling day | 0.13 | 0.06 | 2.15 | **0.034** |

**Supplementary Table 7. Test statistics for the generalized linear model assessing the impact of pH, treatment and day on the diversity indices and the (clr-transformed) abundances of the seven most common bacterial genera (n_pH_information_=134).** dfs=degrees of freedom; Significant results are presented in bold. n=134.

| ***a) Observed ASVs*** | **estimate** | **Std. error** | **p-value** |
| --- | --- | --- | --- |
| pH value | -0.90 | 2.96 | 0.244 |
| treatment | -25.34 | 26.18 | 0.335 |
| day | -0.62 | 1.81 | 0.734 |
| pH value:treatment | 4.62 | 4.23 | 0.278 |
|  |  |  |  |
| ***b) Shannon*** | **estimate** | **Std. error** | **p-value** |
| pH value | -0.31 | 0.97 | 0.050 |
| treatment | -1.91 | 1.39 | 0.174 |
| day | -0.04 | 0.09 | 0.676 |
| pH value:treatment | 0.33 | 0.22 | 0.141 |
|  |  |  |  |
| ***c) PC1 unweighted*** | **estimate** | **Std. error** | **p-value** |
| pH value | 0.11 | 0.06 | 0.079 |
| treatment | 0.42 | 0.54 | 0.430 |
| day | 0.02 | 0.04 | 0.666 |
| pH value:treatment | -0.06 | 0.09 | 0.449 |
|  |  |  |  |
| ***d) PC1 weighted*** | **estimate** | **Std. error** | **p-value** |
| pH value | -0.01 | 0.03 | 0.655 |
| treatment | -0.17 | 0.23 | 0.443 |
| day | -0.05 | 0.02 | **0.002** |
| pH value:treatment | 0.03 | 0.04 | 0.425 |
|  |  |  |  |
| ***e) Weissella*** | **estimate** | **Std. error** | **p-value** |
| pH value | 0.77 | 0.49 | 0.118 |
| treatment | 10.26 | 4.30 | **0.019** |
| day | 0.68 | 0.30 | **0.023** |
| pH value:treatment | -1.65 | 0.70 | **0.019** |
|  |  |  |  |
| ***f) Staphylococcus*** | **estimate** | **Std. error** | **p-value** |
| pH value | 0.09 | 0.86 | 0.917 |
| treatment | -9.94 | 7.56 | 0.191 |
| day | -0.90 | 0.52 | 0.086 |
| pH value:treatment | 1.32 | 1.22 | 0.284 |
|  |  |  |  |
| ***g) Fructobacillus*** | **estimate** | **Std. error** | **p-value** |
| pH value | -0.21 | 0.84 | 0.802 |
| treatment | 1.59 | 7.39 | 0.830 |
| day | 1.72 | 0.51 | **0.001** |
| pH value:treatment | -0.36 | 1.20 | 0.761 |
|  |  |  |  |
| ***h) Corynebacterium*** | **estimate** | **Std. error** | **p-value** |
| pH value | -0.61 | 0.70 | 0.384 |
| treatment | -5.18 | 6.21 | 0.406 |
| day | 0.01 | 0.43 | 0.989 |
| pH value:treatment | 1.03 | 1.00 | 0.308 |
|  |  |  |  |
| ***i) Streptococcus*** | **estimate** | **Std. error** | **p-value** |
| pH value | -2.67 | 0.85 | **0.002** |
| treatment | -12.79 | 7.52 | 0.091 |
| day | -0.19 | 0.52 | 0.708 |
| pH value:treatment | 2.13 | 1.22 | 0.081 |
|  |  |  |  |
| ***j) Actinomyces*** | **estimate** | **Std. error** | **p-value** |
| pH value | -2.80 | 0.81 | **0.001** |
| treatment | -14.44 | 7.20 | **0.047** |
| day | 0.39 | 0.50 | 0.430 |
| pH value:treatment | 2.39 | 1.16 | **0.042** |
|  |  |  |  |
| ***k) Gemella*** | **estimate** | **Std. error** | **p-value** |
| pH value | -0.32 | 0.75 | 0.673 |
| treatment | 2.09 | 6.59 | 0.752 |
| day | -1.21 | 0.45 | **0.009** |
| pH value:treatment | -0.10 | 1.07 | 0.922 |
